# Hair Follicle Generation by Injections of Adult Human Follicular Epithelial and Dermal Papilla Cells into Nude Mice 

**DOI:** 10.22074/cellj.2016.3916

**Published:** 2017-02-22

**Authors:** Mohammadali Nilforoushzadeh, Elham Rahimi Jameh, Fariba Jaffary, Ehsan Abolhasani, Gelavizh Keshtmand, Hajar Zarkob, Parvaneh Mohammadi, Nasser Aghdami

**Affiliations:** 1 Skin and Stem Cell Research Center, Tehran University of Medical Science, Tehran, Iran; 2 Skin Diseases and Leishmaniasis Research Center, Isfahan University of Medical Sciences, Isfahan, Iran; 3 Department of Developmental Biology, University of Science and Culture, Tehran, Iran; 4 Department of Regenerative Biomedicine, Cell Science Research Center, Royan Institute for Stem Cell Biology and Technology, ACECR, Tehran, Iran; 5 Department of Stem Cells and Developmental Biology, Cell Science Research Center, Royan Institute for Stem Cell Biology and Technology, ACECR, Tehran, Iran

**Keywords:** Alopecia, Cell Therapy, Baldness, Dermal Papilla

## Abstract

**Objective:**

Dermal papilla and hair epithelial stem cells regulate hair formation and the growth cycle. Damage to or loss of these cells can cause hair loss. Although several studies claim to reconstitute hairs using rodent cells in an animal model, additional research is needed to develop a stable human hair follicle reconstitution protocol. In this study, we have evaluated hair induction by injecting adult cultured human dermal papilla cells and a mixture of hair epithelial and dermal papilla cells in a mouse model.

**Materials and Methods:**

In this experimental study, discarded human scalp skins were used to obtain dermal papilla and hair epithelial cells. After separation, cells were cultured and assessed for their characteristics. We randomly allocated 15 C57BL/6 nude mice into three groups that received injections in their dorsal skin. The first group received cultured dermal papilla cells, the second group received a mixture of cultured epithelial and dermal papilla cells, and the third group (control) received a placebo [phosphate-buffered saline (PBS-)].

**Results:**

Histopathologic examination of the injection sites showed evidence of hair growth in samples that received cells compared with the control group. However, the group that received epithelial and dermal papilla cells had visible evidence of hair growth. PKH tracing confirmed the presence of transplanted cells in the new hair.

**Conclusion:**

Our data showed that injection of a combination of adult human cultured dermal papilla and epithelial cells could induce hair growth in nude mice. This study emphasized that the combination of human adult cultured dermal papilla and epithelial cells could induce new hair in nude mice.

## Introduction

Millions of men and women who experience emotional stress and psychiatric problems due to hair loss spend considerable amounts of money to find a miracle therapy for hair preservation. Different treatment strategies such as medications and surgical modalities have been introduced for hair loss. Current surgical treatment involves harvesting small pieces of hair follicles from a donor site and grafting them to the bald or thinning areas (1,3). However, an adequate donor supply is critical to a successful transplantation procedure. Drug applications require substantial research prior to approval. 

To this end there are ongoing studies to develop more effective and novel therapies. 

Recent development in cellular and molecular biology of hair and skin have resulted in introduction of cell therapy to this field. Hair stem cells offer new hopes for treatment of alopecia. Hair follicles are the main source of multipotent stem cells in the skin (4). The division and differentiation of stem cells can potentially renew skin and restore hair follicles (5,6). Their fate is controlled by both intrinsic factors and surrounding microenvironment characteristics. Advances in hair cell culture techniques and evaluation of microarray data indicate a close signal interaction between stem cells and the population of mesenchymal cells located in the skin, known as dermal papilla cells (7). 

Dermal papilla cells are specialized mesenchymal cells that reside at the base of the hair follicle which regulate both development and growth of these follicles. In addition, they provide a reservoir of multipotent stem cells (8,9). The receptive property of epithelial stem cells and trichogenicity of dermal papilla cells, both *in vivo* and *in vitro*, has been examined (10,15). Nonetheless, there is no reported protocol for reconstitution of mature human hair follicles. 

The present study aimed to assess the potential of injected cultured adult dermal papilla in induction of hair follicle growth in an animal model. In addition, we evaluated the combination of dermal papilla and epithelial cells in induction of hair follicle growth. 

## Materials and Methods

### Isolation of human hair follicles

In this experimental study, human scalp samples were taken from the occipital skin of volunteer donors during a hair transplant procedure. The Institutional Review Board and Ethical Committee of Royan Institute and Tehran University approved this study and all experimental procedures were performed in accordance with the Declaration of Helsinki. All tissue donors (10 patients) were males with an average age of 30 years (range: 25-35 years). Eligible patients provided written informed consent. A scalp strip of 1.5 cm×1.5 cm was harvested and cut into 1- to 2-mm pieces that contained the hair follicles. Samples were transferred in Dulbecco’s Modified Eagle’s Medium and Ham’s F-12 Nutrient Mixture (DMEM/ Ham’s F-12) supplemented with 10% fetal bovine serum (FBS), 50 μg/mL of L-glutamine (Gibco, Germany), and 50 μg/mL penicillin/streptomycin. Ice packs were placed on top of the samples during transportation. 

### Immunofluorescence staining

Before the epithelial stem cell culture, we assessed the presence of human hair stem cells in the hair samples. Human scalp hairs were stained with CD200 and K15 antibodies. Briefly, the human scalp hair was fixed by paraformaldehyde (Sigma, Germany). The slide mounted sections were deparaffinized and rehydrated. Microwave antigen retrieval was used with 6 mM of citrate buffer (pH=6.8, Sigma, Germany). Slides were washed and treated with blocking reagent followed by overnight incubation with the primary antibodies KRT15 (Abcam1385 LHKRT15, 1:100) and goat anti-human CD200 (R&D Systems, 1:50). After washing, the samples were incubated with secondary antibodies anti-mouse RRX (1:400) and anti goat Alexa-488 (1:400) at room temperature for 1 hour. Nuclei were stained with DAPI (Sigma, Germany) and tissues analyzed by fluorescent microscopy (Nikon, Japan, camera: Axico CAM MRC5) (16). 

### Isolation and culture of epithelial and dermal papilla cells from the hair follicles

The human hair follicles, as sources for the epithelial and dermal papilla cells, were transferred to the laboratory, washed by phosphate-buffered saline (PBS^-^), and soaked in 70% ethanol (Merck, Germany) for 30 seconds. The samples were in- cubated at 4˚C in a solution that contained 1.2 U/ mL of dispase II enzyme (Gibco, Germany) for 16 hours. 

Hairs were pulled out of the dermis. The lower one-third of the hair follicles were cut by the ap- plication of gentle pressure with the tip of a fine needle under a stereomicroscope. These parts were considered as dermal papilla. The area between the skin epidermis and dermal papilla was considered the area that contained follicular epithelial cells (17,18). 

The follicle part that contained the epithelial cells was incubated in a 0.05%-trypsin enzyme solution for 20 minutes. The trypsin enzyme was neutralized by a DMEM/F12 (Gibco, Germany) and 10% FBS (Gibco, Germany) solution, after which the cells were filtrated through a 70-μm pore mesh and centrifuged at 200 g for 5 minutes. The separated epithelial cells were distributed on Matrigel-coated plates (19) and cultured in a medium that contained DMEM/Ham’s F-12 (1:1) supplemented with 10% FBS, 50 μg/ mL L-glutamine (Gibco, Germany), 50 μg/mL penicillin/streptomycin, and a KGM kit (Lonza, Switzerland). The medium was replaced by new medium every three days. After 14 days, confluent cells were washed by PBS^-^ for one to two minutes, and then treated with 0.05% trypsin. We stored the separated primary epithelial cell cultures in 10% FBS/90% dimethyl sulfoxide (DMSO, Sigma, Germany) at -70˚C for one day, and then at -180˚C. We evaluated CD200 expression in the cultured epithelial cells 14 days after culture (20,21). Briefly, cells were fixed, washed with PBS^-^ , and incubated overnight with CD200 antibody (R&D Systems, 1:50) at 4˚C. Then, cells were washed with PBS^-^ and stained with secondary antibodies RRX. Cells were washed again, the nuclei were stained with DAPI and analyzed by fluorescent microscopy (Nikon, Japan) (16). 

In order to culture dermal papilla cells, we incubated the samples that contained dermal papilla in 1 mg/mL of collagenase I enzyme (Sigma, Germany) solution for 4 hours at 37˚C. Then the cells were pipetted, filtrated through a 70 μm pore mesh, and centrifuged for five minutes at 1500 RPM. The separated cells were spread on fibronectin (BD Bio Coat, Belgium)-coated plates in DMEM/Ham’s F-12 (1:1) supplemented with 10% FBS, 50 μg/mL of L-glutamine, and 50 μg/ml penicillin/streptomycin media. We passaged the cells when they reached 90% confluency. The cells reached appropriate confluency after 3 passages during 21 days. The cells were separated, stored in 10% FBS/90% DMSO at -70˚C for one day, and then stored at -180˚C (18,22). 

We confirmed the existence of dermal papilla cells in the culture by detection of alkaline phosphatase and toluidine blue staining of the passage-3 cells. The presence of alkaline phosphatase in dermal papilla cells indicated that these cells could induce hair growth and form colonies in the culture media. In order to detect alkaline phosphatase activity, the cultured dermal papilla cells were seeded on the plate without any coating and fixed, then washed in distilled water and incubated in the developing solution per the Biovision k412- 500 kit instructions. Laminine, type IV collagen, chondroitin 6-sulfate, and heparan sulfate proteoglycans are major components of the dermal papilla extracellular matrix. These materials comprise the basal membrane (23). We have used toluidine blue staining to determine the hyaluronic acid-induced metachromatic characteristics of these cells. The cells were fixed, washed with PBS^-^ , stained with toluidine blue, and examined under a light microscope (18,22). 

### Injection of epithelial and dermal papilla cells into nude mice

We investigated the competencies of the cultured human dermal papilla cells and the dermal papilla and epithelial cell mixture to induce hair follicle formation after transplantation. The culture cell suspensions were injected into C57BL/6 nude mice. We purchased 15 male nude mice, four to six weeks of age that had a mean weight of 100 g from Omid Institute (Omid Institute for Advanced Biomodels, Iran). The mice were kept in cages on a 12-hour light/12-hour dark cycle at 24˚C with access to food and water ad libitum. We randomly divided the mice into three groups (n=5 per group) that received either the suspension of dermal papilla cells, combination of dermal papilla and follicular epithelial cells, or placebo (PBS^-^). 

The mice adapted to standard conditions and food regimen, they were anesthetized under sterile conditions by an intraperitoneal injection of 100 mg/kg of ketamine hydrochloride (Rotexmedica, Germany) and 10 mg/kg of xylazine hydrochloride (Alfasan, Holland). Each mouse received 25 intradermal injections into the skin by a 27-G needle connected to a micro syringe. We injected 1.2×10^6^ passage-3 dermal papilla cells in five mice in one group and a mixture of 0.6×10^6^ epithelial (primary cultured cells) cells with 1.2×10^6^ dermal papilla cells (passage-3) into other five mice in second group. Moreover, the remaining 5 mice just received PBS^-^ as a control group. The injection site was 2×2 cm^2^ and the volume of solution in each transplantation site was 1 ml. The injection site was covered by Mepitel^®^ wound dressing (Molnlycke Health Care) to prevent drying of the experimental area. 

### Tracking injected cells after transplantation

We tracked the fate of the transplanted dermal papilla and hair epithelial cells by cell labeling with PKH26 (Sigma-Red, Germany) per the manufacturer’s instructions. The cells were analyzed by fluorescent microscopy (Nikon, Japan). Briefly, 1.2×10^6^ dermal papilla cells (passage-3) and a mixture of 6×10^5^ hair epithelial cells (primary epithelial cell culture) and 1.2×10^6^ dermal papilla cell (passage-3) suspension were incubated in PKH solution for four minutes at room temperature and then washed to remove unlabeled PKH cells. Frozen sections were prepared, stained with DAPI, and viewed under fluorescence microscopy (Nikon, Japan) in order to observed the PKH labeled cells within the grafts (24). 

### Histological evaluation of hair-inducing properties of transplanted cells

We observed the injection site weekly for hair growth or any reaction to the injection from the first through the fifth weeks after the injections. In order to evaluate the presence of hair in the mice, we obtained a biopsy sample from the treatment area during the fifth week of injection. 

The biopsy samples were fixed in 10% formalin, soaked in paraffin, and sliced into 5 μm thick sections. The sections were stained by hematoxylin and eosin (H&E) and assessed under a light microscope. We prepared and checked all fields in five sections from each mouse under light microscopy. 

## Results

### Human occipital tissue presents epithelial stem cells

**Fig.1:**
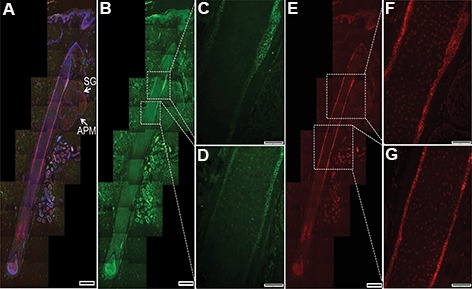
Presence of human hair stem cells in human scalp samples. A. Longitudinal section of human anagen hair showed CD200 and K15 expressions in the outermost (ORS) layer of human hair. CD200 expressed between the insertion point of the arrector pili muscle (APM) and the sebaceous gland (SG). K15 expressed in the bulge and inferior of the bulge area (sub bulge). White arrows show APM, SG, B. CD200 expressed between the APM and SG. Dotted square represents CD200 expression, C, D. Magnified CD200 expression area, E. K15 expressed in the bulge and inferior of the bulge area (sub bulge). Dotted square represents K15 expression, F and G. Magnified K15 expression area. Nuclei were stained with DAPI (scale bar: 50 μm).

We performed immunofluorescence staining on the human hair samples to detect CD200 and K15 expressions. Our results showed that cells between the sebaceous gland (SG) and arrector pili muscle (APM, human hair bulge area) expressed CD200 (Fig .1A-D) and K15 expressed in the bulge and sub-bulge area (Fig .1A,E-G). Therefore, the human hair tissue contained epithelial stem cells. 

### Culture and characterization of dermal papilla and hair epithelial stem cells

**Fig.2:**
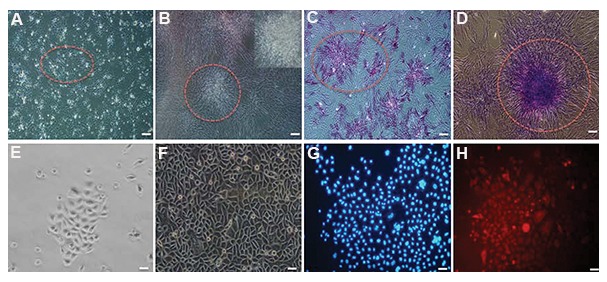
Human dermal papilla and epithelial cell characterization and culture. A. Dermal papilla produced a sunflower colony in culture (dotted circle), B. Upper panel showed magnified colony, C, D. Dermal papilla colony tested strongly positive for alkaline phosphatase and toluidine blue staining (dotted circles) (scale bar: 200 μm), E. Epithelial cells began to proliferate after four days (scale bar: 100 μm), F. Epithelial cells became confluent after 14 days, G and H. CD200 expression detected after culture (Red). Nuclei were stained with DAPI (blue) (scale bar: 200 μm).

Dermal papilla cells attached to the fibronectin surface and began to proliferate. After three to five days, these spindle-shaped cells tended to form colonies with a sunflower pattern (Fig .2A). After two weeks, confluent dermal papilla cells occupied the bottom of the plate and had the capability to be passaged (Fig .2B). We performed alkaline phosphatase staining in order to confirm the nature of these cells. The staining in the third passage indicated the expression of alkaline phosphatase by these cells (Fig .2C). Staining by toluidine blue showed that after the third passage, dermal papilla cells had the capability to produce extracellular matrix (Fig .2D). 

After confirmation of the presence of epithelial stem cells in human hair tissue, we extracted and cultured total epithelial cells. *In vitro* culture of epithelial cells revealed that the epithelial cells began to proliferate slowly after four days (Fig .2E) and generated a compact, small, and confluent epithelial sheet. Within two weeks of culture, the cells proliferated considerably, covered the plate completely, and achieved confluency (Fig .2F). The squamous appearance of cells under light microscopy indicated their epithelial nature. Characterization of epithelial cells by immunofluorescence showed CD200 expression in cultured cells (Fig .2G,H). 

### Generating new human hair using cultured dermal papilla and epithelial cells

A dermatopathologist searched for the presence of hair growth in the prepared biopsy samples from the mice. Histopathologic reports are shown in Table 1. Although histopathologic examination and H&E staining showed evidence of hair growth in all samples that received dermal papilla and the mixture of cells, we observed that mice in the mixture group (epithelial and dermal papilla cells) had hairs that could be seen emerging from the skin.

**Fig.3:**
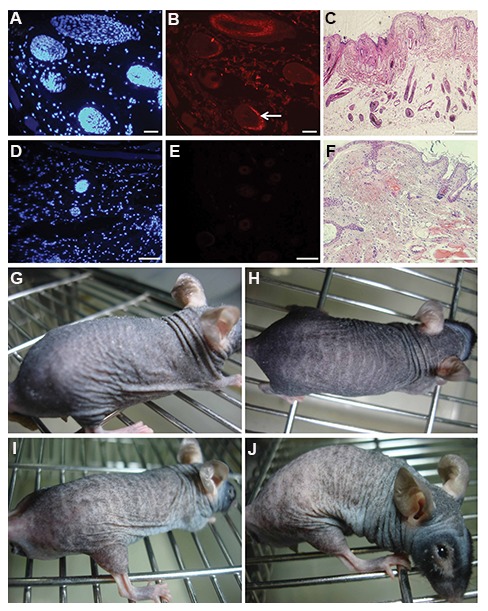
Hair formation ability of human cultured adult dermal papilla cells in nude mice. A, B. Dermal papilla cells labeled with PKH participated in new hair growth in nude mice. Nuclei were stained with DAPI. White arrow showed human cell participation in new hair regeneration (scale bar: 200 μm), C. Hematoxylin and eosin (H&E) staining showed new hairs produced in the dermal papilla group (scale bar: 500 μm), D, E. No PKH+ cells were detected in the control group. Nuclei were stained with DAPI (scale bar: 100 μm), F. H&E staining shows no hair in the control group (scale bar: 500 μm), G-J. Evaluation of nude mice during first and fifth weeks showed no new hair production on the dorsal skin of injected nude mice in the dermal papilla and control groups.

In the mice that received 1.2×10^6^ dermal papilla cells, the histopathologic findings showed evidence of hair growth. PKH staining revealed the existence of injected cells in the grown hairs (Fig .3A, B). Results of H&E staining showed the creation of bud-like structures in the dermis (Fig .3C). There were no PKH+ cells detected in the control group (Fig .3D, E). No hair was seen in H&E stained samples from the control group. Histopathological examination showed few hair follicles in the hypodermis (Fig .3F). Terminal hairs, which were distinguishable on the dorsal skin of the injected mice, were not detected in dermal papilla group (Fig .3G, H). Moreover, no hair was detected in control group (Fig .3I, J).

**Fig.4:**
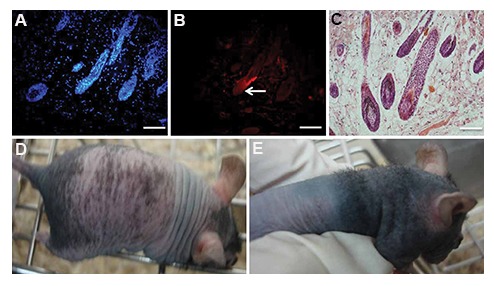
Hair formation ability of cultured adult human dermal papilla and epithelial cells in nude mice. A, B. PKH staining showed that chimeric hairs from human and mouse cells were produced in the dermis. Nuclei were stained with DAPI. White arrow showed human cell participation in new hair regeneration (scale bar: 100 μm), C. Hematoxylin and eosin (H&E) staining showed new hair formation in the dermis (scale bar: 100 μm), D and E. Assessment of nude mice after epithelial and dermal papilla cell injections within first and fifth weeks showed new hair construction at the fifth week.

The majority of hairs were in anagen phase in mice that received a mixture of epithelial and dermal papilla cells. PKH staining revealed that chimeric hairs were made in the dermis (Fig.4A, B). H&E staining showed new hair creation in contrast to the control group (Fig .4C). We observed new hair growth after the cell injections on the backs of nude mice at the injection site (Fig .4D, E). There was no evidence of any tumors according to H&E staining in any of the groups.

**Table 1:** Histopathologic results of injecting human adult cultured dermal papilla and mixture of epithelial and dermal papilla cells to nude mice


Group	Mice no.	Histopathologic report

Dermal papilla cells	1	Many follicles were in anagen phase but they were progressing to catagen phase.
	2	Most of the follicles were in anagen phase but they were progressing to catagen phase.
	3	There were many hair follicles in anagen and catagen phases.
	4	There were many hair follicles in anagen and catagen phases.
	5	There were many hair follicles in anagen and catagen phases.
Mixed epithelial and dermal papilla cells	6	There were many hair follicles in anagen and catagen phases with high proliferation rate.
	7	Increase in cells in pre-anagen and anagen phases with high proliferation rate.
	8	Follicles were seen in early anagen phase.
	9	Follicles were seen in early anagen phase.
	10	Follicles were seen in early anagen phase.
Control group	11	Few hair follicles in hypodermis; increase in connective tissue and fibroblasts. Most of the hair follicles were in catagen and telogen phase.
	12	Few hair follicles in hypodermis; increase in connective tissue and fibroblasts. Most of the hair follicles were in catagen and telogen phase.
	13	Hair follicles were surrounded by sebaceous glands without maturation toward terminal hairs.
	14	Few hair follicles in hypodermis; increase in connective tissue and fibroblasts. Most of the hair follicles were in catagen and telogen phase.
	15	Few hair follicles in hypodermis; increase in connective tissue and fibroblasts. Most of the hair follicles were in catagen and telogen phase.


## Discussion

Over the last decade, major advances have been made in both understanding and treatment of hair loss. At least three distinct breakthroughs increased hope and interest for future research: development of selective 5α-reductase inhibitors, development of specific cell culture systems, identification of the physiology of the hair follicle bulge, and their reservoir of stem cells. These last two developments have made the hair transplantation process possible with the development of specific cell culture systems as the subject of recent studies of combined medical and surgical treatments (25,26). 

The hair follicle bulge serves as an excellent reservoir of epithelial stem cells (27). This fascinating structure has been already identified in limited areas such as the bottom of the intestinal crypts, the limbal zone of the cornea, and the subventricular zone of the brain (7). In theory, several steps are needed for successful hair follicle generation under *in vitro* conditions: isolation of epithelial and dermal papilla cell populations, expansion of their numbers by culture, maintenance of the proliferative and inductive properties of each cell linage, and the provision of exogenous signals to enhance their interaction. However, the final trichogenic ability should be assessed in an *in vivo* model. 

In this study, we attempted to isolate, culture, and test the ability of epithelial stem and dermal papilla cells to induce hair growth in an *in vivo* model. Immunofluorescence staining showed that the human hair scalp expressed both CD200 and K15; hence, epithelial stem cells could be isolated and cultured *in vitro*. Regarding CD200 expression in bulge area (between the SG and insertion of the APM), we selected CD200 for more analysis. Isolation of epithelial cells and evaluation of CD200 expression after culture showed the presence of hair stem cells. Expression of alkaline phosphatase by cultured dermal papilla cells showed inductive properties of these cells *in vitro*. Toluidine blue showed that after the third passage, dermal papilla cells had the capability to produce extracellular matrix. 

Although injection of mixed dermal papilla and epithelial cells led to hair growth in mice in our experiment, none of the mice that only received dermal papilla cells had visible hair growth. It seemed that while dermal papilla cells could not induce detectable hair follicles when injected alone, they assisted with hair growth when mixed with epithelial cells. 

Inamatsu et al. (14) evaluated the ability of rat embryonic and adult dermal papilla cells in inducing hair growth on a piece of rat’s sole skin. Both cultured dermal papilla cells and an intact dermal papilla between the epidermis and dermis resulted in the generation of hair follicles. They concluded that epidermal cells reprogram embryonic processes when exposed to embryonic dermal papilla cells, whereas they directly initiate anagen of the hair cycle when they receive stimulatory signals from adult rat dermal papilla cells. 

Aoi et al. (15) evaluated the ability of rat dermal papilla cells to induce hair follicle growth in five transplantation methods *in vivo*. They observed new hair follicle regeneration in the rat’s sole skin regardless of the transplantation method. However, the hemi-vascularized sandwich (HVS) method was superior in terms of number and maturity of follicles, which was attributed to direct epithelial- mesenchymal signaling and better vascularization/ oxygenation. 

Several possible explanations exist for the observed discrepancy between reported results. First, the inductive ability of dermal papilla cells might decrease after multiple passages. However, two recent developments were associated with increased hair-inductive activity of cultured dermal papilla cells: first, the co-culture of epidermal keratinocytes with dermal papilla cells prolonged the hair-inductive property of dermal papilla cells. This feature was mostly attributed to unknown soluble factors secreted by epidermal keratinocytes, which maintained the hair-inductive capability of dermal papilla cells during their population doublings in culture. Second, Wnt signaling from external source resulted in a preserved hair- inductive capability of dermal papilla cells (28). Although alkaline phosphatase and toluidine blue staining showed trichogenic properties of our cultured dermal papilla cells, additional factors might be needed to confirm trichogenicity of these cells in the culture. Second, the culture condition was another possible explanation for reduced trichogenicity. However, Kang et al. (29) evaluated the hair-inducing capacity of dermal papilla cells in three-dimensional (3D) spheroid cultures. Mixtures of mouse epidermal cell with two-dimensional (2D) - or 3D-cultured dermal papilla cells were implanted into nude mice and compared with implantation of epidermal cells alone. Only combined mouse epidermal and mesenchymal implantation induced new hair follicle formation. Interestingly, various 3D dermal papilla passages resulted in hair follicle formation, while no new hair follicles were obtained with the same 2D cultures. 

## Conclusion
